# Six RNA binding proteins (RBPs) related prognostic model predicts overall survival for clear cell renal cell carcinoma and is associated with immune infiltration

**DOI:** 10.17305/bjbms.2021.6097

**Published:** 2021-08-22

**Authors:** Qianwei Xing, Jiaochen Luan, Shouyong Liu, Limin Ma, Yi Wang

**Affiliations:** 1 Department of Urology, Affiliated Hospital of Nantong University, Nantong, Jiangsu Province, China; 2 Department of Urology, The First Affiliated Hospital of Nanjing Medical University, Nanjing, Jiangsu Province, China

**Keywords:** RNA-binding proteins, overall survival, clear cell renal cell carcinoma, model, prognosis

## Abstract

The aim of this article was to construct an accurate prognostic model using RNA-binding proteins (RBPs) to predict overall survival (OS) for patients with clear cell renal cell carcinoma (ccRCC) as well as to reveal its associations with immune infiltration. Expression profiles based on RBPs and clinical follow-up parameters were obtained from the Cancer Genome Atlas (TCGA) and the ArrayExpress databases. Through univariate COX and LASSO regression analyses, the RBPs based signature was developed. A total of six RBPs (CLK2, IGF2BP2, RNASE2, EZH2, PABPC1L, and RPL22L1) were eventually used to establish a prognostic signature. Based on this signature, ccRCC patients were classified into high-risk and low-risk subgroups and significant OS was obtained in both the internal and external datasets (*p* < 0.05). AUCs of its ROC curve were all above 0.70 and this signature was an independent prognostic factor of OS for ccRCC (*p* < 0.05). Nomograms were also constructed to visualize the relationships among individual predictors and 1-, 3-, and 5-year OS for ccRCC. Furthermore, the established RBPs based signature was strongly related to critical clinicopathologic characteristics such as grade (*p* = 8.921e−12), stage (*p* = 1.421e−11), M (*p* = 1.662e−05), and T stage (*p* = 7.907e−10). Moreover, 12 kinds of tumor-infiltrating immune cells were significantly linked to high-risk and low-risk groups classified by our constructed model (all *p* < 0.05). Our study successfully identified six RBPs as a robust prognostic signature in ccRCC by both external and internal verifications. Besides, our established model displayed significant associations with immune infiltration. In addition to original clinical parameters, our findings may further help clinicians in predicting patients’ survival status and creating individualized treatment plans.

## INTRODUCTION

Renal cell carcinoma (RCC) is estimated to have caused approximately 76,080 new cases as well as 13,780 new deaths in the United States in 2021 alone [1]. Accounting for approximately 70-80% of RCC, clear cell renal cell carcinoma (ccRCC) comprises the majority of cancer deaths [2,3]. Due to the resistance to radiotherapy and chemotherapy, the surgical resection is recommended as the primary therapy for ccRCC by the clinical guidelines [4,5]. Despite the tremendous progress in novel diagnostic tools and early surgical treatment, the cancer metastasis of ccRCC is still extremely common and 2 years survival rate of metastatic patients is <20% [6]. Therefore, new diagnostic markers and therapeutic targets are urgently required to understand the potential molecular mechanism and predict the disease occurrence, progression, and metastasis for these cases.

RNA-binding proteins (RBPs), also known as proteins interacting with different types of RNAs (ncRNAs, rRNAs, miRNAs, snRNAs, tRNAs, mRNAs, and snoRNAs), are recommended as pivotal post-transcriptional regulators not only regulating the spatiotemporal expression of genes but also modulating the disease pathogenesis [7]. Accounting for 7.5% of about 20,500 protein-coding genes in humans [8], RBPs blind to RNA or constitute crucial components of ribonucleoprotein (RNPs) to participate in RNA metabolism [9]. Till now, genome-wide screening of the human genome has identified more than 1500 RBPs that played an essential role in biogenesis, surveillance, transport, localization, and degradation of RNA in line with the genetic and biochemical studies [8,10-12]. According to target RNA categorization, it is found that 50% of RBPs involve pathways of mRNA metabolism, 11% of them establish ribosomal proteins, and the others associate with different kinds of non-coding RNA metabolism [8,13].

With the discovery of non-coding RNAs and increasing understanding of post-transcriptional regulation in tumors, cancer-related RBPs are employed to construct highly intricate regulatory networks. Moreover, the disturbance of these networks is likely to have a relationship with primary carcinogenic hits, increasing aggressiveness, and accelerating progression [14]. Accumulating data have underscored that RBPs primarily alter various cancer-associated downstream targets to exert influence on carcinogenesis and development. Researches as UNR in melanoma [15], LARP1 in ovarian cancer (OC) [16], IMP3 in leukemia [17], QKI in kidney cancer [18], LIN28B as well as MSI in colon cancer [19], and IMP2 in glioma [20] have been applied to disclose the RBP cancer-specific post-transcriptional networks. However, the roles of most RBPs have not yet been found in tumors, and the functions of RBPs in the progress of cancer remain relatively unexplored. In the current research, our efforts were made to establish a RBPs based signature to predict overall survival (OS) for ccRCC. Our results were anticipated to help clinician predict patients’ survival status and to promote the specific individualized treatment than original clinical parameters.

## Materials and Methods

### Identification of expression profiles and differentially expressed RBPs (DERBPs) from public databases

Expression profiles based on RBPs together with clinical follow-up parameters were obtained from The Cancer Genome Atlas (TCGA, https://portal.gdc.cancer.gov/). As detailed in Supplementary Table S1, the census of human RBPs was obtained from the article of “A census of human RNA-binding proteins” in Nature Reviews Genetics [8]. The raw data were pre-handled by the “limma” package of R software and standardized by log2 transformation. In addition, to screen DERBPs between ccRCC tumor tissues and adjacent normal kidney tissues, log2|fold change (FC)| ≥1 and false discovery rate (FDR)<0.05 were set as the cutoff criterion. Moreover, the external validation cohort (E-MTAB-1980 dataset) was obtained from the ArrayExpress dataset (https://www.ebi.ac.uk/arrayexpress/).

### Establishment and validation of the risk score model

To explore the associations between OS and DERBPs in the training database and testing database, the univariate Cox regression analysis was applied and *p* = 0.05 was set as the cutoff value. Then, LASSO Cox regression method was utilized to establish the model based on prognostic RBPs. LASSO regression could fit the generalized linear model, contributing to variable selection and regularization. Subsequently, the risk score algorithm was constructed as following:


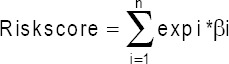


Therein, βi represented the regression coefficient of each gene, and expi represented the expression level of each gene.

### Nomogram construction and validation

According to the independent prognostic clinical parameters and our established RBPs signature, a novel nomogram was conducted to forecast the likelihood of OS for ccRCC. To estimate the accuracy of the nomogram, ROC curves as well as the area under the ROC curve (AUC) was plotted. Moreover, calibration curves were generated from the “rms” package of R software, and applied to compare the observed and predicted results of this nomogram. Similarly, the methods were employed in the external validation set to verify these outcomes.

### Verification of the mRNA expression and prognosis utilizing the ICGC, GEO datasets, and Kaplan–Meier plotter website

International Cancer Genome Consortium (ICGC) dataset cohort (http://dcc.icgc.org) and two Gene Expression Omnibus (GEO) datasets (https://www.ncbi.nlm.nih.gov/geo/; GSE14994 and GSE6344) were utilized to verify the mRNA expression of these six hub RBPs. Kaplan–Meier plotter online tool (http://kmplot.com/analysis/) was employed to evaluate the prognosis of hub RBPs in ccRCC cohorts [21].

### Quantitative real-time PCR (qRT-PCR)

We followed the manufacturer’s instructions and qRT-PCR was utilized to verify the mRNA expression of these six hub RBPs in four pairs of ccRCC tumor and adjacent normal kidney tissues acquired from Affiliated Hospital of Nantong University by means of StepOne Plus RT-PCR system (Applied Biosystems, Foster City, CA, USA). Our used primers were displayed as following: Actin (F: 5’-ATGACTTAGTTGCGTTACACC-3’, R: 5’-GACTTCCTGTAACAACGCATC-3’);

CLK2 (F: 5’-GGGGAGTTACCGTGAACACTA-3’, R: 5’-CGTGTCCGGTCACTACTACTTG-3’);

EZH2 (F: 5’-GTACACGGGGATAGAGAATGTGG-3’, R: 5’-GGTGGGCGGCTTTCTTTATCA-3’);

IGF2BP2 (F: 5’-AGCCTGTCACCATCCATGC-3’, R: 5’-CTTCGGCTAGTTTGGTCTCATC-3’);

PABPC1L (F: 5’-AACATCTACGTGAAGAACCTCCC-3’, R: 5’-CACTCAGCATTTTCCCAAACTG -3’);

RNASE2 (F: 5’-TGTGGTAACCCAAATATGACCTG-3’, R: 5’-GGTCTCGTCGTTGATCTCTGT-3’);

RPL22L1 (F: 5’-GCAATTTCTACGGGAGAAGGTT-3’, R: 5’- ACTCGAAGCCAATCACGAAGA-3’);

### Validation of the protein expression utilizing the Human Protein Atlas (HPA) database and CPTAP analysis

Using the HPA online database (http://www.proteinatlas.org/), the protein expression of the hub RBPs in ccRCC was validated by immunohistochemical (IHC) staining. As detailed in HPA database, CLK2 was stained by HPA055366 antibody in IHC; EZH2 was stained by CAB009589 antibody in IHC; IGF2BP2 was stained by HPA035145 antibody in IHC; RNASE2 was stained by HPA044983 antibody in IHC; RPL22L1 was stained by HPA056207 antibody in IHC; whereas PABPC1L immunohistochemistry outcomes had not been provided yet. Scale bar for each IHC picture was 200 um. We also utilized the UALCAN website (http://ualcan.path.uab.edu/analysis-prot.html) to validate the protein of the hub RBPs expression between the primary ccRCC tumor and normal tissues by Clinical Proteomic Tumor Analysis Consortium (CPTAC) dataset analysis [22].

### Tumor-infiltrating immune cells (TIICs) estimation

The expressions of TIICs in every ccRCC sample from the TCGA dataset were calculated as previously described [23]. Based on our established model, ccRCC patients were divided into a high- and low-risk group. Through R packages, we could explore whether or not TIICs were linked to these two groups, under the threshold of *p* < 0.05.

### Statistical analysis

Statistical analysis was accomplished by utilizing the R software 3.6.3. Student’s t-test or Wilcoxon rank-sum test was utilized for continuous variables and Chi-squared test or Fisher’s exact test was employed for categorical variables. Kaplan–Meier survival curves as well as the log-rank test were implemented utilizing the “survival” package of R software. The ROC curves were plotted using the R package “survival ROC.” For the whole statistical analyses, *p* values were two sided and its values below 0.05 were regarded to be significantly different.

### Availability of data and material

RNA sequencing data of ccRCC, together with clinical follow-up parameters, were got from the Cancer Genome Atlas (TCGA) database and the ArrayExpress database (E-MTAB-1980). ICGC dataset and two GEO datasets (GSE14994 and GSE6344) were utilized to verify the mRNA expression of these six hub RBPs.

## RESULTS

### RBPs based expression profiles and differently expressed RBPs identification

**FIGURE 1:**
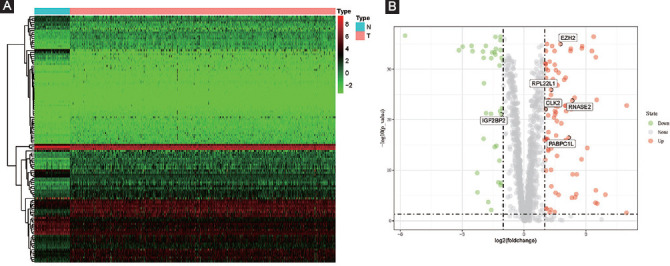
One hundred and twenty-five differentially expressed RNA-binding proteins get from TCGA ccRCC cohort; (A) heatmap; N=adjacent normal renal tissues; T=ccRCC tumor tissues; scale=FPKM values of gene expression; (B) volcano plot.

**SUPPLEMENT FIGURE S1:**
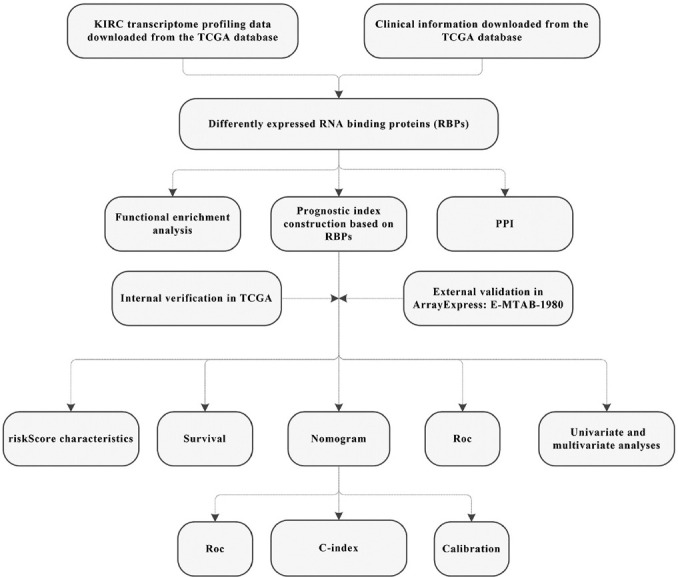
The whole workflow chart of this study.

The whole workflow of this study is shown in Supplementary Figure S1. RNA sequencing data of ccRCC and the clinicopathological characteristics including 72 adjacent normal renal tissues and 539 ccRCC tumor samples, were obtained from the TCGA cohort. When selecting |log2(FC)|>1 and FDR<0.05 as the threshold, 125 differently expressed RBPs were screened out from a list of 1542 RBPs, including 38 downregulated and 87 upregulated RBPs (Supplement Table S2). The expression heatmap of 125 differently expressed RBPs and the volcano plot of all RBPs are demonstrated in Figure 1A and 1B.

### Prognostic model (risk score) construction

**FIGURE 2:**
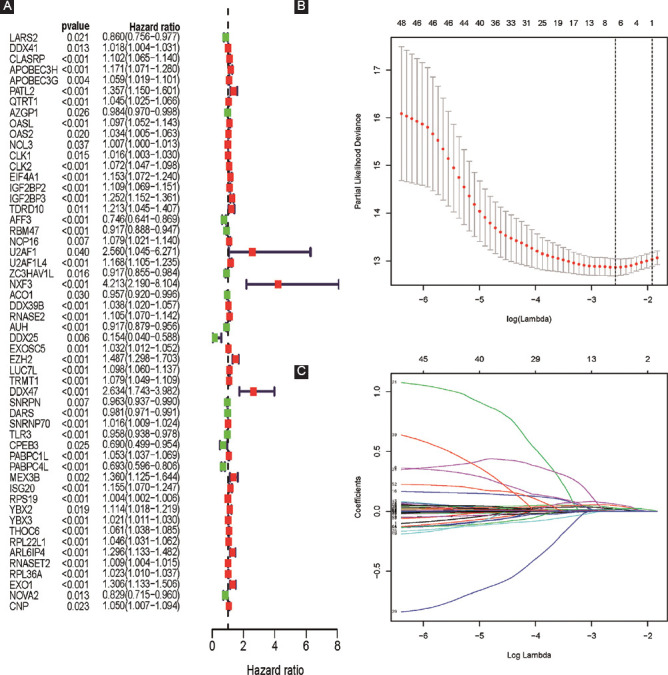
Prognostic model index (risk score) construction based on univariate Cox regression analysis and LASSO analysis; (A) the forest plot of 54 differentially expressed RNA-binding proteins (RBPs) screened out by univariate Cox regression; (B-C) LASSO coefficient profiles of the prognostic RBPs.

Based on the univariate Cox regression analysis, 54 candidate RBPs were identified (Figure 2A). Then, the LASSO Cox regression model was performed and six vital prognostic RBPs including CLK2, IGF2BP2, RNASE2, EZH2, PABPC1L, and RPL22L1 were finally selected (Figure 2B-C and Table 1). As a result, a six prognostic RBPs signature was constructed and the risk score of each sample was calculated: Risk score = (0.01812 × ExpCLK2) + (0.02605 × ExpIGF2BP2) + (0.04622 × ExpRNASE2) + (0.05813 × ExpEZH2) + (0.01903 × ExpPABPC1L) + (0.01191 × ExpRPL22L1).

**TABLE 1:**
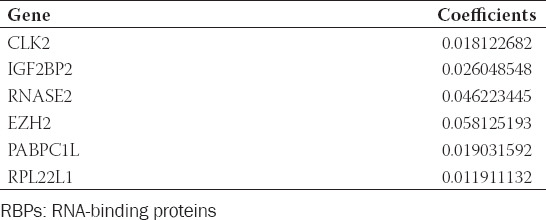
Coefficients of these six key prognostic RBPs

### Evaluation, external and internal verification of six RBPs based signature (risk score)

**FIGURE 3:**
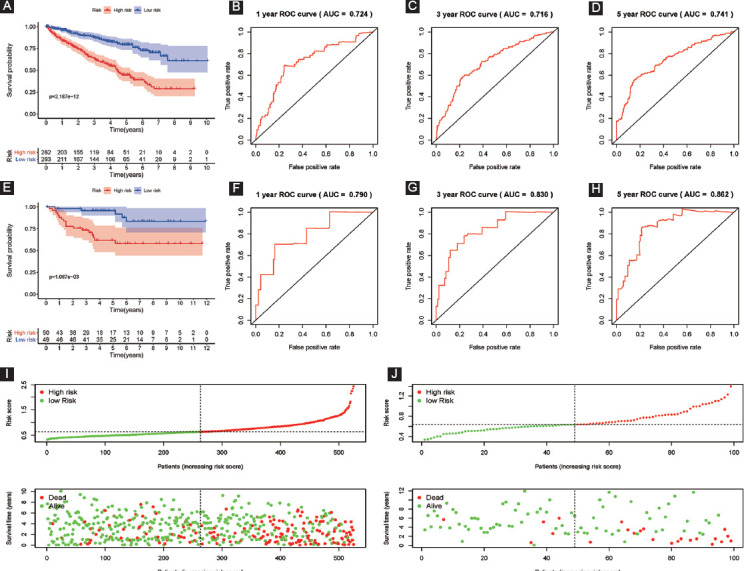
Evaluation and external verification of our established signature; (A) Kaplan–Meier survival curves of OS in the whole training dataset (TCGA); (B) 1-year ROC in the whole training dataset (TCGA); (C) 3-year ROC; (D) 5-year ROC; (E) Kaplan–Meier survival curves of OS in the external validation dataset (ArrayExpress); (F) 1-year ROC in the external validation dataset (ArrayExpress); (G) 3-year ROC; (H) 5-year ROC; (I-J) the distribution of risk scores for each sample and patients’ survival status in the whole training dataset (TCGA) and the external validation dataset (ArrayExpress); therein, A-D and I represented the whole training dataset (TCGA); E-H and J represented the external validation dataset (ArrayExpress).

Based on six RBPs established signature (risk score), patients with ccRCC were classified into two groups (high- and low-risk groups), and KM survival analysis shed light on that patients with low risks had a much better OS than those with high risks (*p* = 2.187e−12, Figure 3A). To better evaluate our established model, the ROC curve and its AUC were further analyzed. Our results indicated that 1-, 3-, and 5-year AUC values were 0.724, 0.716, and 0.741, separately, showing superior predictive veracity of patients’ survival results (Figure 3B-D and Table 2). In addition, when the risk score increased, patients would have more dead events (Figure 3I).

**TABLE 2:**
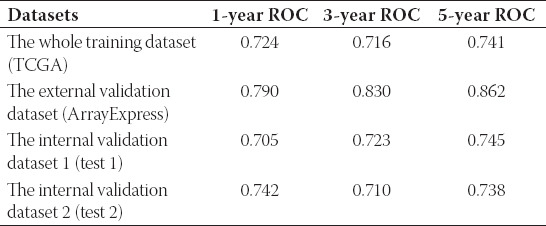
External and internal verification datasets of 1-year, 3-year, and 5-year ROC

**FIGURE 4:**
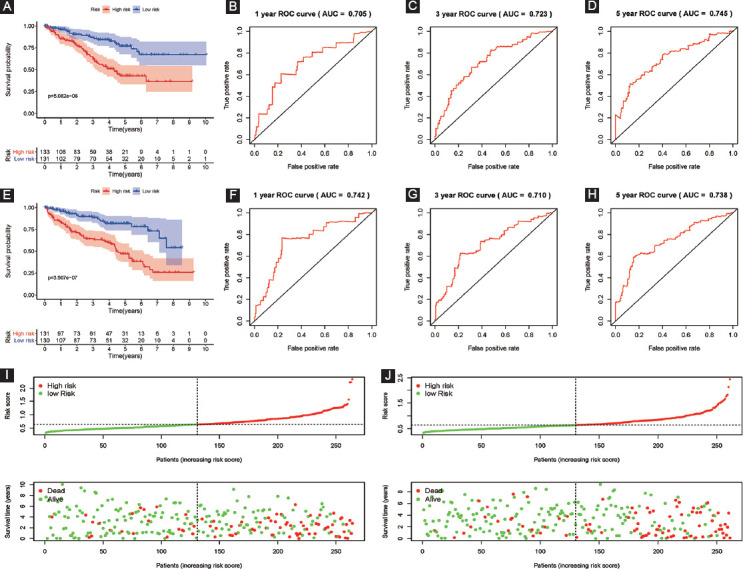
Internal verification of six RNA-binding proteins established signature; (A) Kaplan–Meier survival curves of OS in the internal validation dataset 1 (test 1); (B) 1-year ROC in the internal validation dataset 1 (test 1); (C) 3-year ROC; (D) 5-year ROC; (E) Kaplan–Meier survival curves of OS in the internal validation dataset 2 (test 2); (F) 1-year ROC in the internal validation dataset 2 (test 2); (G) 3-year ROC; (H) 5-year ROC; (I-J) the distribution of risk scores for each sample and patients’ survival status in the internal validation dataset 1 (test 1) and in the internal validation dataset 2 (test 2); therein, A-D and I represented the internal validation dataset 1 (test 1); E-H and J represented the internal validation dataset 2 (test 2).

The external validation database (E-MTAB-1980, n = 99), the internal validation dataset 1 (n = 264), and the internal validation dataset 2 (n = 261) were utilized as validation databases to verify our signature. In terms of Kaplan–Meier survival analysis, all three validation sets showed similar outcomes (the external validation dataset: *p* = 1.067e−03; the internal validation dataset 1: *p* = 5.082e−06; and the internal validation dataset 2: *p* = 3.507e−07; Figure 3E, Figure 4A and E). ROC analysis displayed that the AUC for 1-year, 3-year, and 5-year OS of these three databases were all above 0.70 (Figure 3F-H, Figure 4B-D, Figure 4F-H, Table 2). Figure 3J and Figure 4I-J displayed that patients would have more dead events, when the risk score increased. In a word, our established model possessed superior sensitivity and specificity in predicting OS for ccRCC.

### Our established six RBPs based signature could serve as an independent prognostic parameter for OS

In the univariate Cox analysis, the high-risk groups revealed a 5.411-fold, 4.453-fold, 6.776-fold, and 57.907-fold increased risk of death than those in the low-risk groups (the whole training dataset (TCGA): 95% CI 3.897-7.512; the internal validation dataset 1: 95% CI 2.824-7.023; the internal validation dataset 2: 95% CI 4.142-11.084; and the external validation dataset: 95% CI 10.449-320.911, all *p* < 0.001; respectively). By means of multivariate Cox analysis, the six RBPs based model was strongly associated with OS for patients with ccRCC in the whole training database: HR = 3.417, 95% CI 2.259-5.168; in the internal validation dataset 1: HR = 3.377, 95% CI 1.896-6.012; and in the internal validation dataset 2: HR = 3.585, 95% CI 1.774-7.246, all *p* < 0.001; except for the external validation dataset: HR = 2.055, 95% CI 0.149-28.250; *p* = 0.590) (Table 3).

**TABLE 3:**
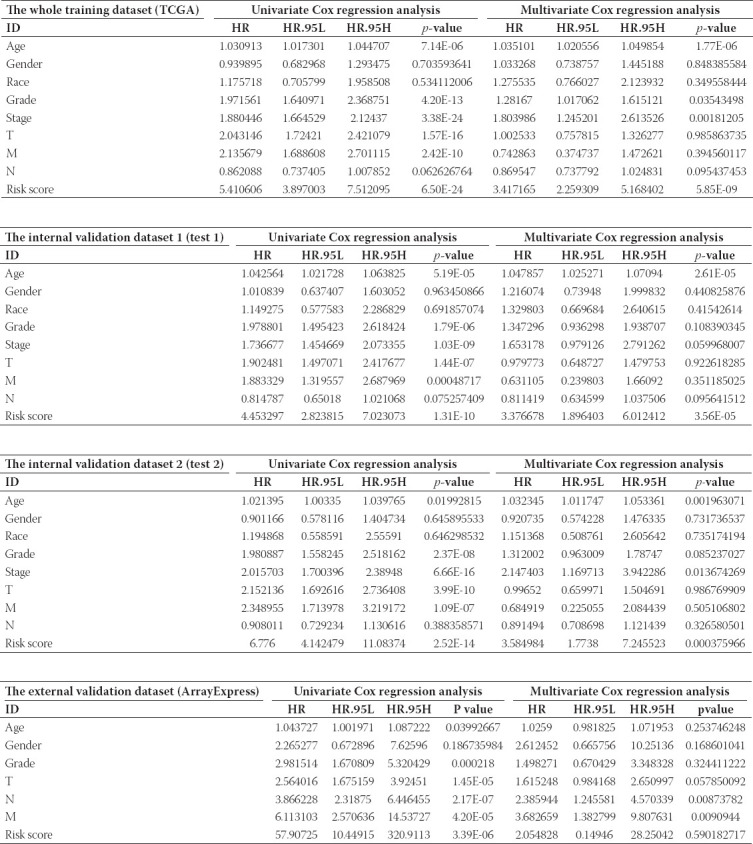
Univariate and multivariate Cox regression analysis of external and internal verification datasets for overall survival (OS)

### Construction of the novel nomogram on the basis of clinical characteristics and the signature

**FIGURE 5:**
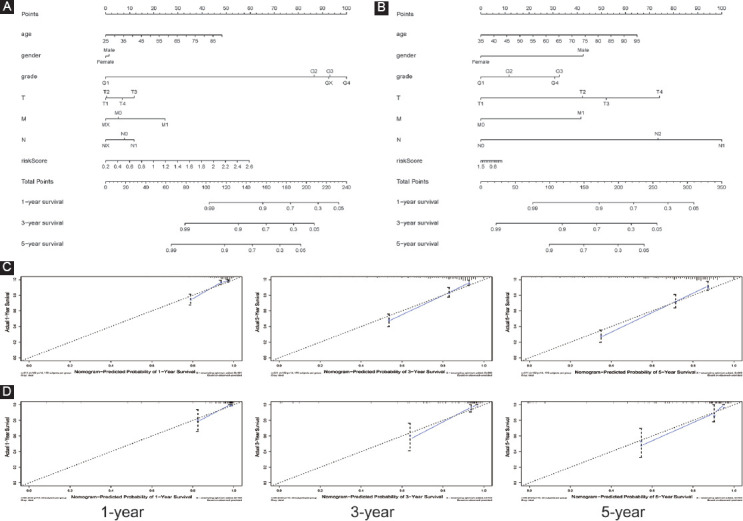
Nomogram and calibration plots in both TCGA and ArrayExpress databases; (A-B) nomogram in the TCGA and ArrayExpress databases, respectively; (C-D) calibration plot of 1-, 3-, and 5-year OS prediction in the TCGA and ArrayExpress databases, separately.

**SUPPLEMENT FIGURE S2:**
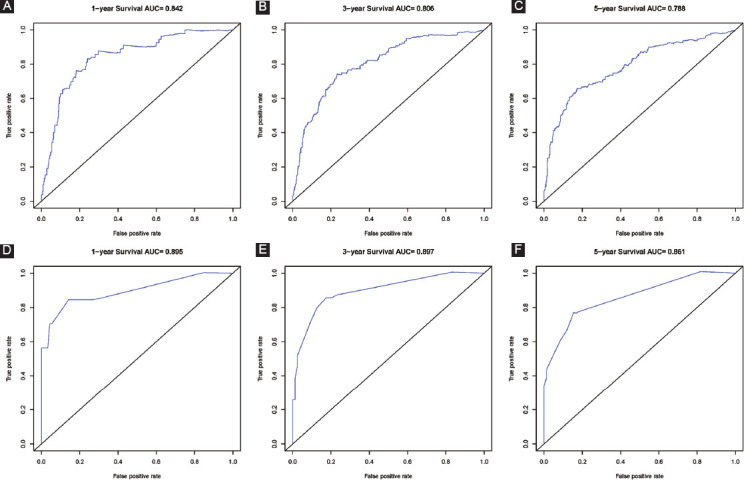
One-, 3-, and 5-year ROC analyses of nomogram in both TCGA and ArrayExpress databases (E-MTAB-1980); (A-C) 1-, 3-, and 5-year in TCGA dataset; (D-F) 1-, 3-, and 5-year in ArrayExpress dataset (E-MTAB-1980).

To provide a quantitative method to predict the ccRCC patients’ prognosis in clinical trials, we established a compound nomogram in both the TCGA and ArrayExpress databases. Our outcomes presented that this novel nomogram could better predict OS of patients (Figure 5A) and its 1-year, 3-year, and 5-year AUC values and C-index in the TCGA dataset were 0.842, 0.806, 0.788, and 0.79, respectively, showing an excellent prognostic ability (Table 4 and Supplement Figure S2). In the calibration curve, the diagonal line represented the most ideal outcome; the closer the predictive values were to the diagonal line, the more consistent they were with the actual situation. Calibration plots of this nomogram revealed that the predictive values were significantly similar to the ideal ones (Figure 5C). We also built another prognostic nomogram in the ArrayExpress dataset (E-MTAB-1980) as an external validation set to verify the previous results (Figure 5B). Its 1-, 3-, and 5-year AUC values and C-index in the ArrayExpress dataset were 0.895, 0.897, 0.861, and 0.872, separately, showing a better predictive accuracy in OS (Table 4 and Supplement Figure S2). Calibration plot also displayed the satisfactory conformity between the predicted and actual values (Figure 5D).

**TABLE 4:**
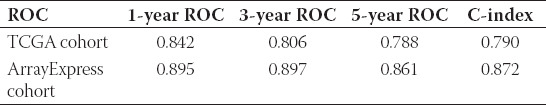
1-year, 3-year, and 5-year ROC and C-index of nomogram for in TCGA and ArrayExpress datasets

### Association between these six prognostic RBPs, risk score, and clinicopathologic characteristics

**SUPPLEMENT FIGURE S3:**
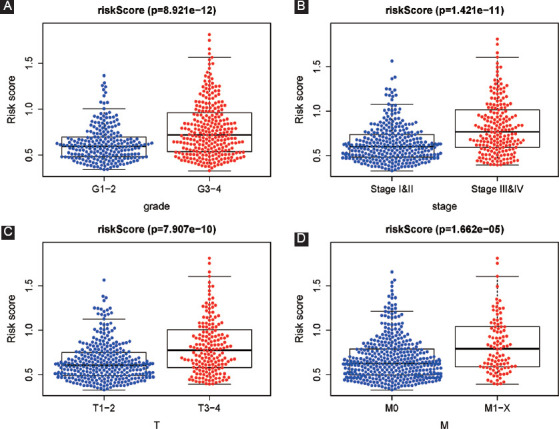
Relationships between clinicopathologic characteristics and our established risk score; distribution of risk scores in (A) grade; (B) stage; (C) T stage; (D) M stage.

The relationships between clinicopathologic characteristics, risk score, and six prognostic RBPs were explored. Our results revealed that the six RBPs based signature (risk score) was firmly related to grade (*p* = 8.921e−12), tumor stage (*p* = 1.421e−11), M stage (*p* = 1.662e−05), and T stage (*p* = 7.907e−10) (Supplement Figure S3). In addition, the correlation between six hub RBPs and clinical features was also analyzed (Table 5).

**TABLE 5:**
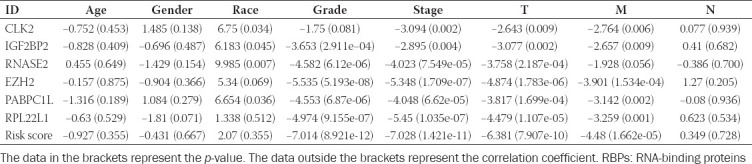
Clinical correlation analysis between these six prognostic RBPs, our established risk score and clinical features

### Validation of the mRNA expression and the prognosis of six RBPs in ccRCC

**FIGURE 6:**
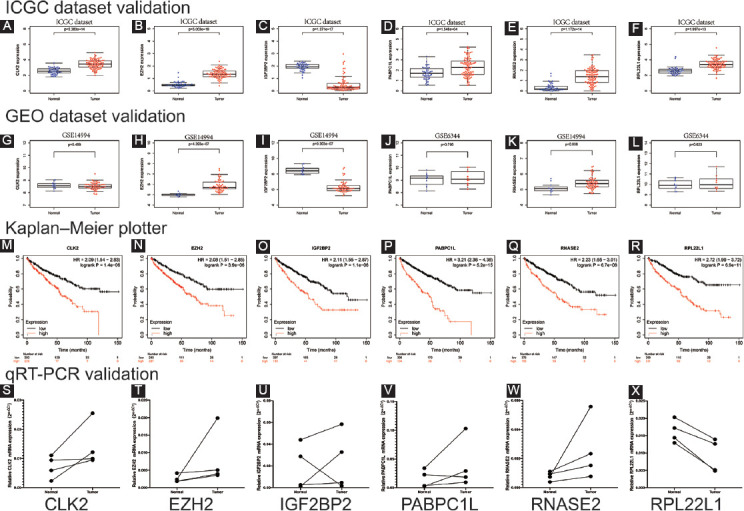
Validation of the mRNA expression and prognostic value of six critical RNA-binding proteins (RBPs) in ccRCC; boxplot of six critical RBPs mRNA expression in ICGC dataset (N=45; T=91); (A) CLK2; (B) EZH2; (C) IGF2BP2; (D) PABPC1L; (E) RNASE2; (F) RPL22L1; boxplot of six critical RBPs mRNA expression in GSE14994 dataset (N=11; T=59) and GSE6344 dataset (N=10; T=10); (G) CLK2, (H) EZH2, (I) IGF2BP2, (J) PABPC1L, (K) RNASE2, (L) RPL22L1; Scale=Gene count values; Kaplan–Meier plotter of (M) CLK2, (N) EZH2, (O) IGF2BP2, (P) PABPC1L, (Q) RNASE2, (R) RPL22L1; quantitative real-time PCR validation of (S) CLK2, (T) EZH2, (U) IGF2BP2, (V) PABPC1L, (W) RNASE2, (X) RPL22L1 mRNA expressions in clinical ccRCC samples (N=4; T=4).

ICGC dataset (http://dcc.icgc.org), containing 45 normal renal and 91 tumor samples, was applied to verify the mRNA expression of these six RBPs (CLK2, EZH2, IGF2BP2, PABPC1L, RNASE2, and RPL22L1). As displayed in Figure 6A-F, they were differentially expressed in tumors compared with normal tissues (all *p* < 0.001). Results from GSE14994 and GSE6344 datasets showed that EZH2, IGF2BP2, and RNASE2 had significant expressions in tumors compared with normal tissues, while the others did not (all *p* < 0.01; Figure 6G-L). KM plotter displayed that six RBPs were remarkably related to OS in ccRCC patients (all *p* < 0.001; Figure 6M-R). qRT-PCR was employed to validate the mRNA expression of these six hub RBPs in four pairs of ccRCC tumor samples and adjacent normal kidney tissues. Based on our results, only RPL22L1 showed significant results (*p* = 0.0067). *p* values of CLK2, EZH2, IGF2BP2, PABPC1L, and RNASE2 were all above 0.054. This might be due to the relatively small number of samples (Figure 6S-X).

### Verification of the protein expression of the critical RBPs in ccRCC

**FIGURE 7:**
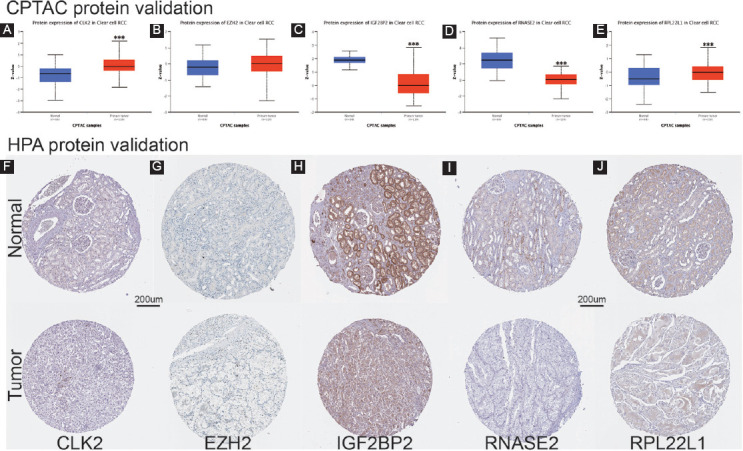
Verification of the protein expression of the critical RNA-binding proteins (RBPs) in ccRCC; boxplot of six critical RBPs protein expression by Clinical Proteomic Tumor Analysis Consortium analysis (A) CLK2; (B) EZH2; (C) IGF2BP2; (D) RNASE2; (E) RPL22L1; immunohistochemistry outcomes from Human Protein Atlas database (F) CLK2; (G) EZH2; (H) IGF2BP2; (I) RNASE2; (J) RPL22L1; scale bar=200 um; ***p < 0.001.

Due to the absence of PABPC1L protein in CPTAC and HPA datasets, only five proteins of CLK2, EZH2, IGF2BP2, RNASE2, and RPL22L1 were analyzed. As presented in Figure 7A-E, these RBPs were differently expressed in ccRCC tumor samples compared with adjacent normal kidney tissues (all *p* < 0.001), except for EZH2 (*p* = 0.962). Besides, immunohistochemistry outcomes were used to validate the protein expression of these hub RBPs (Figure 7F-J). Antibody HPA055366 staining for CLK2 in normal kidney tissue was medium, whereas it was low in tumor tissue. Antibody CAB009589 staining for EZH2 in normal kidney tissue was not detected, whereas it was low in tumor tissue. Antibody HPA035145 staining for IGF2BP2 in normal kidney tissue was low, whereas it was medium in tumor tissue. Antibody HPA044983 staining for RNASE2 in normal kidney tissue was medium, whereas it was not detected in tumor tissue. Antibody HPA056207 staining for RPL22L1 in normal kidney tissue was medium, whereas it was low in tumor tissue. Scale bar for each IHC picture was 200 um.

### Clinical factors stratified by our established signature for OS

**FIGURE 8:**
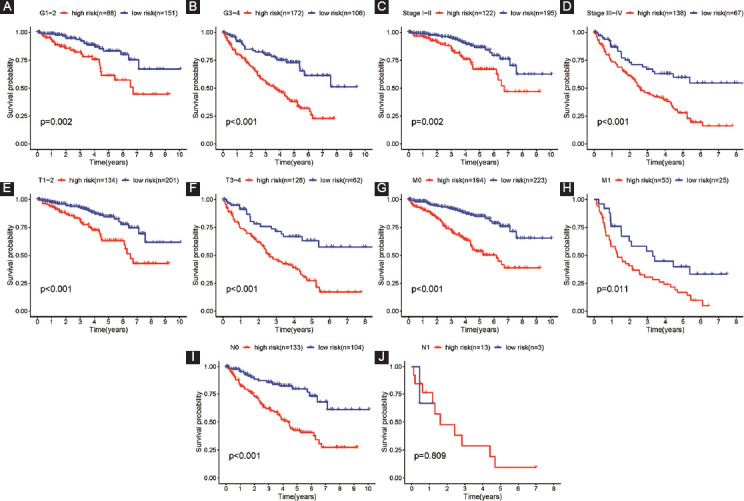
Clinicopathological parameters stratified by risk score for OS; (A) Grade 1-2 stratified by risk score for OS; (B) Grade 3-4; (C) Stage I-II; (D) Stage III-IV; (E) T1-2 stage; (F) T3-4 stage; (G) M0; (H) M1; (I) N0; (J) N1.

According to our established model, ccRCC patients were further divided into subgroups for five clinical factors (stage, grade, N, T, and M). Our results shed light on that except for N1 (*p* = 0.809), our signature was able to predict OS in Grade 1-2, Grade 3-4, Stage III-IV, Stage I-II, T3-4 stage, T1-2 stage, N0, M0, and M1 ccRCC patients (all *p* < 0.05; Figure 8).

### TIICs stratified by our established model

**FIGURE 9:**
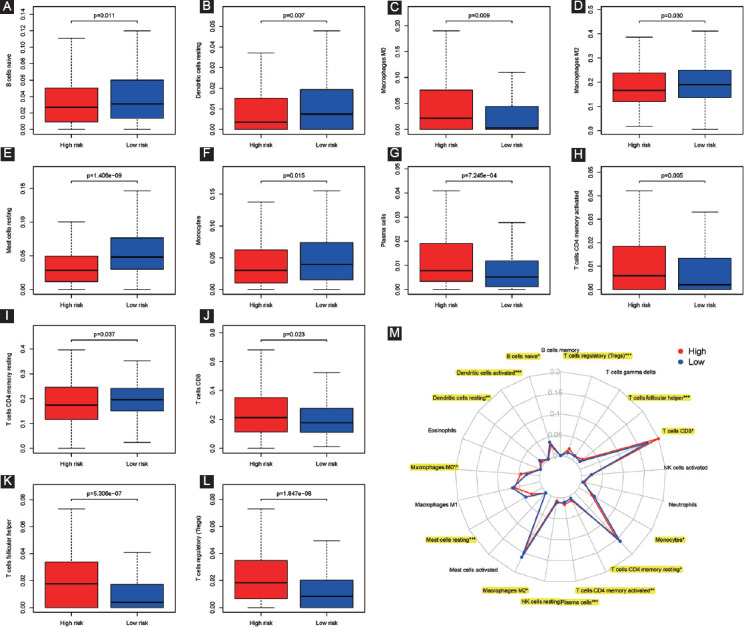
TIICs stratified by our established model; (A) B cells naive distribution; (B) dendritic cells resting; (C) macrophages M0; (D) macrophages M2; (E) mast cells resting; (F) monocytes; (G) plasma cells; (H) T cells CD4 memory activated; (I) T cells CD4 memory resting; (J) T cells CD8; (K) T cells follicular helper; (L) T cells regulatory (Tregs); (M) radar chart.

As detailed in Figure 9A-L, 12 out of 21 TIICs (dendritic cells resting, B cells naive, macrophages M2, macrophages M0, monocytes, mast cells resting, T cells CD4 memory activated, plasma cells, T cells CD8, T cells CD4 memory resting, T cells regulatory (Tregs), and T cells follicular helper) were all significantly stratified by our established model (all *p* < 0.05). Figure 9M summarizes all of the 21 TIICs in high-risk and low-risk groups stratified by our established model by radar chart.

## DISCUSSION

In line with the latest cancer statistics reported from the World Health Organization, the occurrence rate of RCC dramatically increased over the past few decades and it was estimated to have over 140,000 ccRCC-related death events per year [24]. Therefore, it was important to identify reliable biomarkers for better predicting ccRCC patients’ survival. With the development of cancer precision medicine, a variety of signatures or biomarkers have been established for predicting prognosis and therapeutic benefits. Studies illustrated that under the guidance of biomarkers, response rates seen with targeted agents have reached approximately 30%, which were much higher than that of chemotherapy [25]. As reported, RBPs dysregulation happens in the genesis and development of various malignant tumors [14,26,27]. Nevertheless, pivotal functional roles of most RBPs in human cancer remain unclear [8,14]. Moreover, few studies focused on the roles of RBPs in ccRCC’s survival prediction. Based on the ccRCC data from the TCGA set, 125 differently expressed RBPs between kidney and ccRCC tissues were selected. In addition, we adopted univariate COX and LASSO regression analysis to identify hub RBP genes and to build a prognostic signature. This work might contribute to identifying new effective biomarkers for the prognosis of ccRCC.

By means of univariate Cox and LASSO regression analysis, we singled out six hub RBPs (CLK2, IGF2BP2, RNASE2, EZH2, PABPC1L, and RPL22L1). Several studies demonstrated these RBPs played important roles in tumorigenesis and development, even in kidney cancer [14,28,29]. CLK2 could serve as an oncogene in breast cancer, whereas downregulation of CLK2 could suppress tumor growth [30]. Furthermore, CLK2 acted a pivotal part in the control of cell cycle and prognosis of glioblastoma by regulating FOXO3a/p27 pathways [31]. As for IGF2BP2, it was reported that IGF2BP2 was differentially expressed in pancreatic cancer, and its upregulation promotes cancer cells’ growth through stimulating the PI3K/Akt pathway [32]. Wan et al. figured out that RNASE2 was identified as the valuable prognostic predictor in ccRCC patients and utilized to explore the occurrence mechanisms of renal carcinoma and to design individualized treatments for patients [33]. EZH2, namely, the enzymatic subunit of polycomb repressive complex 2 [34,35], has been found to be of great importance in various cancers, including bladder cancer, breast cancer, prostate cancer, and so on [36-38]. Due to the ability of suppressing its enzymatic function, EZH2 became an anti-cancer therapy target and might bring a substantial therapeutic advance in clinical trials [39,40]. Regarding PABPC1L, Wu et al. demonstrated that PABPC1L suppressed migration and cell proliferation in colorectal cancer (CRC), and the expression of PABPC1L in CRC was highly associated with age, pathologic node, pathologic metastasis, pathologic stage, and death [41]. With regard to RPL22L1, Wu et al. demonstrated that RPL22L1 induced epithelial-to-mesenchymal transition in OC and was critical in triggering cell metastasis and maintaining the aggressive phenotype of OC [42].

Next, we established the six RBPs signatures (risk score). The survival curves revealed that ccRCC patients in high-risk groups were associated with poor OS in the training cohort (TCGA) and three validation cohorts (E-MTAB-1980; internal validation cohort dataset 1; and internal validation cohort dataset 2). ROC analysis displayed that our six RBP genes based signature had a moderate performance for predicting ccRCC patients’ OS. Moreover, our established signature could be an independent prognostic parameter of OS for ccRCC. It was worth noting that the *p*-value in the external validation dataset was more than 0.05 in the multivariate Cox regression analysis for the possible reason that its sample sizes were not large enough. Thus, it was acknowledged that the risk score had superior specificity and sensitivity in predicting ccRCC patients’ OS.

In addition, a novel nomogram was constructed that integrated the risk score and several clinical factors (grade, gender, age, T, M, and N) to predict the ccRCC patients’ OS. As for the calibration plot, very excellent outcomes were found in the TCGA cohort between the predicted and actual values. Similarly, a satisfactory agreement was also observed in the external validation dataset. In all, the novel nomogram might better help clinician predict ccRCC patients’ survival status, improve risk stratification, and provide the individualized treatment than before.

In the study, we comprehensively explored the relationship between six RBPs, risk score, and different clinical factors in ccRCC. The results presented that the six RBPs based signature (risk score) was strongly associated with grade, tumor stage, M and T stage, and we also found that risk score would rise when clinicopathological factors (grade, tumor stage, M, and T stage,) increased. Taken together, RBPs might likely have malignant pathological implications in ccRCC, and these discoveries could provide novel insights into the underlying mechanism of RBPs in the progression of cancer. What’s more, through characterizing RBP expression in ccRCC or risk score, novel therapeutic targets could be developed and survival could be predicted for ccRCC patients. A growing number of studies indicated that tumor immune infiltration played key roles in tumorigenesis and tumor progression, having an effect on immunotherapy [43-45]. Hence, in this article, we aimed to identify the associations between our established signature and tumor immune infiltration. The outcomes of us found that 12 out of 21 TIICs (dendritic cells resting, B cells naive, macrophages M2, macrophages M0, monocytes, mast cells resting, T cells CD4 memory activated, T cells regulatory (Tregs), plasma cells, T cells CD8, T cells CD4 memory resting, and T cells follicular helper) were all significantly linked to high-risk and low-risk groups, indicating that immune infiltration was significantly related to our established model for ccRCC patients.

The strength of the article was that our established RBPs related signature was successfully established and evaluated in the other three validation sets (ArrayExpress cohort, the internal validation dataset 1, and the internal validation dataset 2). Moreover, six hub RBPs mRNA or protein expression were also validated by the ICGC, GEO, CPTAP, HPA datasets, and qRT-PCR verification, making our results more persuasive. However, several limitations should also be mentioned. First, our research was retrospective, thus the veracity and availability of six RBPs based signatures should be tested in other public databases, even in the prospective research. Second, two datasets (TCGA and E-MTAB-1980) were screened out of the study with no complete clinical parameters, which might decrease the statistical reliability of multivariate Cox regression analysis. Third, more clinicopathological characteristics were required to be fetched into the novel prognostic nomogram, and additional biomarkers were needed to explore and identify. Fourth, in regard to the expression of IGF2BP2 mRNA, there are many reasons for the difference between the public database and our experiment. For example, the patient samples in the TCGA database are White, Asian, and Black or African-American, and most of them are white. However, in our study, all of the patients are Asian. In addition, the transcriptome profiling data from the TCGA cohort were the RNA-seq data, while the transcriptome profiling data from the GEO cohort were the microarray data which were produced with Illumina HumanHT-12 V4.0 Array, and in our study, we validated the expression level of IGF2BP2 mRNA by qRT-PCR. Besides, the tumor samples consisted of many mixed components. Therefore, there may be a discrepancy about the expression levels of several genes. Moreover, the study revealed that our six RBPs prognostic signature was significantly associated with the OS in ccRCC. However, it was merely analyzed and evaluated by data mining. Hence, more attention should be paid to uncover their roles in ccRCC by functional experiments. Despite the deficiencies described above, the predictive value of the signature in ccRCC patients could not be ignored. In the future, well-designed and multi-institutional studies were still required to verify our findings.

## CONCLUSION

Taken together, our results successfully singled out six critical RBPs (CLK2, IGF2BP2, RNASE2, EZH2, PABPC1L, and RPL22L1) as a robust prognostic signature in ccRCC by external and internal verification, helping clinician predict patients’ survival status. Moreover, this signature could also be an independent prognostic factor for ccRCC. Besides, our established model displayed significant associations with immune infiltration. Further prospective studies were required to verify our established signature and to understand the roles of these six RBPs.

## Figures and Tables

**SUPPLEMENT TABLE 1:**
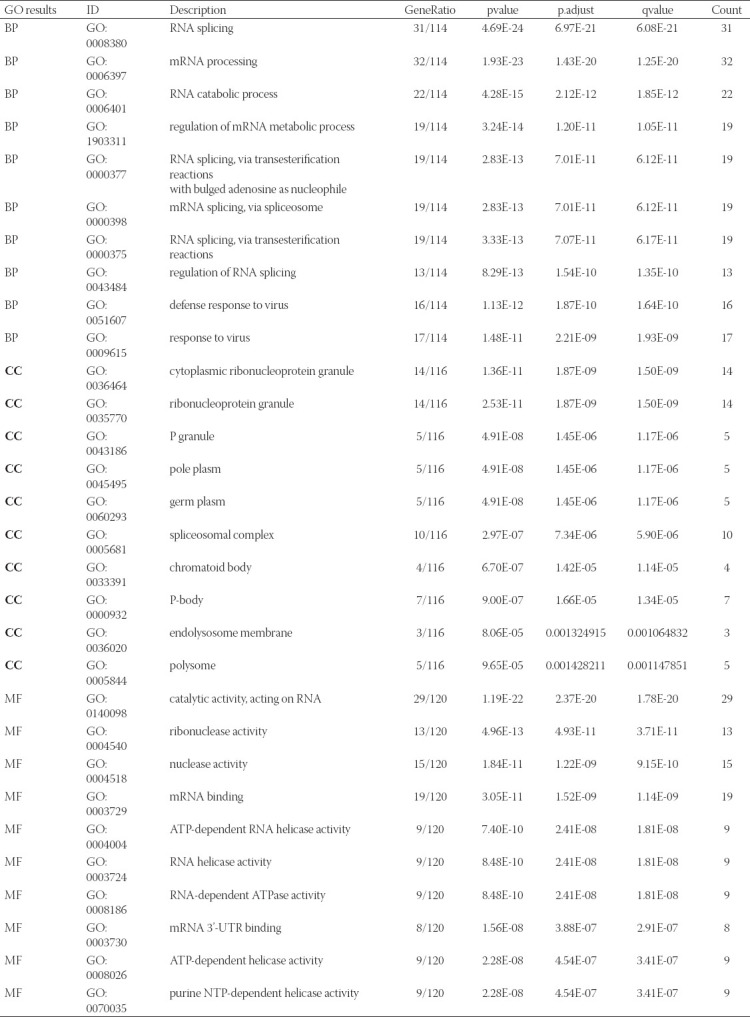
GO results of differently expressed RBPs;

**SUPPLEMENT TABLE 2:**
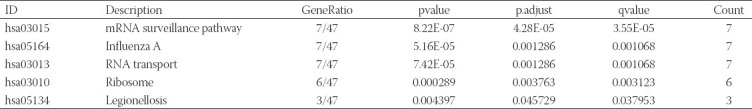
KEGG results of differently expressed RBPs;
